# Using the Multidimensional Health Locus of Control Scale Form C to Investigate Health Beliefs About Bladder Cancer Prevention and Treatment Among Male Patients: Cross-Sectional Study

**DOI:** 10.2196/43345

**Published:** 2023-08-16

**Authors:** Zhaoquan Xing, Meng Ji, Yi Shan, Zhaogang Dong, Xiaofei Xu

**Affiliations:** 1 Department of Urology, Qilu Hospital of Shandong University Ji'nan China; 2 The University of Sydney Sydney Australia; 3 School of Foreign Studies, Nantong University Nantong China; 4 Department of Clinical Laboratory, Qilu Hospital of Shandong Universit Ji'nan China; 5 Center for Reproductive Medicine，Department of Obstetrics and Gynecology, Qilu Hospital of Shandong University Ji'nan China

**Keywords:** health beliefs, Multidimensional Health Locus of Control, Chinese translation, bladder cancer prevention and treatment, male patients, latent class analysis

## Abstract

**Background:**

Bladder cancer is a leading cause of death among Chinese male populations in recent years. The health locus of control construct can mediate health status and outcomes, and it has proven helpful in predicting and explaining specific health-related behaviors. However, it has never been used to investigate health beliefs about bladder cancer prevention and treatment.

**Objective:**

This study aimed to classify male patients into different latent groups according to their beliefs about bladder cancer prevention and treatment and to identify associated factors to provide implications for the delivery of tailored education and interventions and the administration of targeted prevention and treatment.

**Methods:**

First, we designed a four-section questionnaire to solicit data: section 1—age, gender, and education; section 2—the communicative subscale of the All Aspects of Health Literacy Scale; section 3—the eHealth Literacy Scale; and section 4—health beliefs about bladder cancer prevention and treatment measured by the Multidimensional Health Locus of Control Scale Form C. We hypothesized that the participants’ health beliefs about bladder cancer prevention and treatment measured in section 4 could be closely associated with information collected through sections 1 to 3. We recruited 718 Chinese male patients from Qilu Hospital of Shandong University, China, and invited them to participate in a web-based questionnaire survey. Finally, we used latent class analysis to identify subgroups of men based on their categorical responses to the items on the Multidimensional Health Locus of Control Scale Form C and ascertained factors contributing to the low self-efficacy group identified.

**Results:**

We identified 2 subgroups defined as low and moderate self-efficacy groups representing 75.8% (544/718) and 24.2% (174/718) of the total sample, respectively. Men in the low self-efficacy cluster (cluster 1: 544/718, 75.8%) were less likely to believe in their own capability or doctors’ advice to achieve optimal outcomes in bladder cancer prevention and treatment. Men in the moderate self-efficacy cluster (cluster 2: 174/718, 24.2%) had distinct psychological traits. They had stronger beliefs in their own capability to manage their health with regard to bladder cancer prevention and treatment and moderate to high levels of trust in health and medical professionals and their advice to achieve better prevention and treatment outcomes. Four factors contributing to low self-efficacy were identified, including limited education (Year 6 to Year 12), aged ≥44 years, limited communicative health literacy, and limited digital health literacy.

**Conclusions:**

This was the first study investigating beliefs about bladder cancer prevention and treatment among Chinese male patients. Given that bladder cancer represents a leading cause of death among Chinese male populations in recent years, the low self-efficacy cluster and associated contributing factors identified in this study can provide implications for clinical practice, health education, medical research, and health policy-making.

## Introduction

### Background

Bladder cancer is one of the most prevalent tumors and a critical cause of tumor-induced death worldwide [1]. Over 60% of all bladder cancer cases and half of all 165,000 bladder cancer deaths occur in low-income regions worldwide [2]. Three-fourths of all bladder cancer cases occur in men [3]. According to Globocan 2012, bladder cancer ranks 12th among the top 20 most common cancers in China [4]. This cancer is 5 to 6 times more prevalent in men than in women in China [5]. Owing to its high morbidity and mortality rate, bladder cancer has attracted close attention from scientists [6]. A study has reported the causes of developing bladder cancer, including genetic mutations and external risk factors such as tobacco smoking, carcinogen exposure, chlorination of drinking water, and possibly cyclophosphamide [7]. On the basis of the examination of these external factors, we hypothesized that the prevention and treatment of bladder cancer could be affected by another essential factor, that is, patients’ attitudes, which should be investigated to deliver relevant interventions. On the basis of this hypothesis, we raised the following research questions: How many clusters can the study participants be grouped into based on their attitudes toward the prevention and treatment of bladder cancer, specifically their beliefs about the source of reinforcements for health-related behaviors if they develop this cancer? and Can such beliefs be associated with the participants’ demographic features and health literacy status?

Patients’ attitudes toward their conditions can be measured by the locus of control (LOC) construct, as extensively demonstrated by a large variety of pathologies [8-13]. It has been clinically observed that the LOC construct can mediate health status and outcomes [14-16], and it has proven helpful in predicting and explaining specific health-related behaviors [17]. On the basis of the LOC construct, individuals can be categorized into 2 main classes: those believing that their health status (or sickness) results from their own behaviors (*health-internals*) and those considering that their health status is generally determined by factors over which they have poor control, such as chance or powerful others (*health-externals*) [18]. Wallston et al [19] later proved the importance of separately assessing beliefs in the influence of chance and powerful others. More recently, Wallston et al [20] have shown that it is also helpful to distinguish between expectancies related to doctors and those related to significant others (eg, relatives and friends) within the “powerful others” construct. Health LOC has been conceptualized as a construct comprising at least 3 dimensions [19]. The most extensively used and validated instrument of LOC in health is the Multidimensional Health Locus of Control (MHLC) Scale [19]. This measure consists of 18 items rated on a 6-point Likert scale ranging from strongly disagree (n=1) to strongly agree (n=6). These 18 items are divided into three 6-item subscales that measure “Internal,” “Chance,” and “Powerful Others” LOC. Higher scores for each subscale indicate greater belief in that subscale domain in relation to health. There are 3 refined condition-specific versions of the MHLC Scale, namely, the MHLC Form A, the MHLC Form B, and the MHLC Form C [20]. The MHLC Form C consists of 4 subscales, “internal” (6 items), “chance” (6 items), “powerful other people” (3 items), and “doctors” (3 items) [19].

The MHLC Form C has been applied to enhance the knowledge about the point of view of patients with HIV on their complex health condition [21]; to determine how LOC relates to health care use, medication adherence, missed school, and readiness for transition to adult medical care for youth with chronic conditions [22]; and to explore the relationship between LOC and pregnancy [23-27]. Thus informed, we believe that this scale is most likely to solicit patients’ attitudes toward the prevention and treatment of bladder cancer based on which tailor-made education, intervention, and treatment could be delivered for the benefit of the prevention and treatment of this disease. However, it has never been used in this respect. Considering the magnitude of bladder cancer among men in China, it is imperative to examine patients’ attitudes toward and beliefs about the prevention and treatment of this disease to reduce its prevalence and the associated male mortality rate.

### Objective

We aimed to classify male patients into different latent groups according to their attitudes toward and beliefs about bladder cancer prevention and treatment and to identify associated factors to provide implications for the delivery of tailored education and interventions and the administration of targeted prevention and treatment.

## Methods

### Overview

First, we designed the questionnaire. We then recruited male patients from the Qilu Hospital of Shandong University, China, who were invited to complete the web-based questionnaire. Finally, we used latent class analysis (LCA) to identify subgroups of men based on their categorical responses to items on the MHLC Form C.

### Questionnaire Design

We designed a four-section questionnaire (Multimedia Appendix 1), including section 1: age, gender, and education; section 2: the “Communicative Health Literacy” subscale of the All Aspects of Health Literacy Scale (AAHLS) [28]; section 3: the eHealth Literacy Scale (eHEALS) [29]; and section 4: the MHLC Form C [19]. The “Communicative Health Literacy” subscale is defined as more advanced skills to actively participate in everyday activities, extract information, derive meaning from different forms of communication, and apply new information to changing situations [28]. The 8-item eHEALS evaluates study participants’ knowledge and skills that are essential for using eHealth resources and interventions [29]. It does not have subscales. Literacy in health information is becoming a critical factor essential for health status [30]. The 18-item MHLC Form C comprises 4 subscales that measure “internal,” “chance,” “doctor,” and “powerful others” LOC, that is, beliefs that the source of reinforcements for health-related behaviors is primarily internal, a matter of chance, or under the control of doctors or powerful others, respectively [19]. Such beliefs can motivate health behavior, which refers to taking voluntary actions to promote health, reduce health risks [31], and mediate health status [15,32]. Individuals categorized as having an “internal” LOC are more likely to engage in health behaviors and are more knowledgeable about their health problems [33,34]. Considering that the MHLC Form C is a “general purposes, condition-specific locus of control scale that could easily be adapted for use with any medical or health-related condition” [20], we adapted it for use with bladder cancer in the questionnaire. Informed by relevant studies [15,30-34], we hypothesized that the participants’ status of health belief and self-confidence measured by the MHLC Form C in section 4 could be closely associated with information collected through sections 1-3.

### Participant Recruitment and Questionnaire Survey

The study participants were recruited from the Qilu Hospital of Shandong University, China, using randomized sampling. Participants who had met four predefined inclusion criteria were invited to participate in this survey: (1) being aged ≥18 years, (2) having at least primary education (Year 6 schooling) to understand the questions in the questionnaire, (3) being patients rather than relatives accompanying patients, and (4) participating in the survey voluntarily. We made face-to-face contact with Mandarin Chinese–speaking patients who were attending the outpatient clinic and those who were hospitalized to identify those who satisfied the inclusion criteria, explain to them the purpose of the survey, and ask them to participate in the web-based survey as scheduled. We identified 988 eligible patients.

The survey lasted 1 month, from July 20 to August 19, 2022. The questionnaire was administered via *wenjuanxing* [35], the most popular web-based questionnaire platform in China. Participants filled out the administered questionnaire on the web. Returned questionnaires were considered valid only when all question items included were answered according to our predefined validation criterion. On August 20, 2022, the returned questionnaires were downloaded in the format of an Excel file (Microsoft Corp) from *wenjuanxing*. Out of 988 patients, a total of 718 (72.7% response rate) answered questionnaires were returned. We double-checked the returned questionnaires and found all of them to be valid.

### Data Collection, Coding, and Analysis

On August 20, 2022, the answered questionnaires were downloaded in the format of an Excel file from *wenjuanxing* (Multimedia Appendix 2). We double-checked the validity of the returned questionnaires before coding valid data using the predefined coding schemes (Multimedia Appendix 2) based on Likert scales with varying score ranges for different questionnaire items. Subsequently, we used LCA (Latent GOLD 5.0) to identify subgroups of men based on their categorical responses to the items on the MHLC Form C and to ascertained factors contributing to the low self-efficacy group identified.

LCA is increasingly being applied in social and health sciences. LCA has methodological advantages over traditional clustering techniques [36-39]. A notable benefit of LCA is the probabilistic attribution of latent class membership to study participants using maximum likelihood estimation [36]. Therefore, each observed participant attained a probability of belonging to a certain latent class. For example, within a 2-class LCA solution, a study participant can have 2 probabilities associated with either latent class. On the basis of the conditional independence assumption of LCA, the combined probabilities of class memberships sum to 1, based on the conditional independence assumption of LCA. The probabilistic nature of LCA adds to the complexity of interpreting the results. However, in practice, the more flexible, intuitive approach of LCA when compared with “hard, rigid” clustering techniques allows researchers more insights into the impact of predictor variables on latent class membership fluidity and dynamics, and the susceptibility of class memberships to the definition and selection of probability thresholds to suit different research purposes.

### Ethics Approval and Participation

This study was approved by the Ethics Review Board of the Qilu Hospital of Shandong University, China (KYLL-202208-026). The study data were anonymized to protect the privacy and confidentiality of the study participants. As the participants voluntarily participated in the survey to support and promote academic research, no compensation was provided for them as per the common practice in China.

## Results

### Descriptive Statistics

Table 1 presents the descriptive statistics of the data collected from the patient participants. All data in the 718 returned questionnaires were valid. The patients had a mean age of 46.41 (SD 10.143) years. In total, 718 patients were men. The mean score for education was 2.89 (SD 1.398), indicating that their average educational level was between Year 9 and Year 12. The mean scores of the 3 communicative items on the AAHLS were 1.84 (SD 0.763), 1.91 (SD 0.751), and 1.95 (SD 0.744) for item 1, item 2, and item 3, respectively. These mean scores indicate that they “sometimes” needed help to read and comprehend health information and complete official documents and were “sometimes” able to identify and secure others’ help. The scores for the 8 items on the eHEALS ranged from 2.68 (SD 1.200) to 2.86 (SD 1.239), indicating their uncertainty about their skills to use eHealth resources and interventions. As with their scoring performance on the MHLC Form C, they scored on average 18.05 (SD 4.390), 16.80 (SD 4.435), 9.75 (SD 3.333), and 8.70 (SD 2.757) on the “internal,” “chance,” “doctor,” and “powerful others” subscales, respectively. The determined response of “slightly disagree” for the “internal” subscale indicates that they somehow did not believe in their internal drives to maintain health. The determined response between “moderately disagree” and “slightly disagree” for the “chance” subscale implies that they were generally less likely to attribute their health to a matter of luck. The determined response between “slightly disagree” and “slightly agree” for the “doctor” subscale means that they were generally uncertain about the role of doctors in the maintenance of their own health. The determined response between “moderately disagree” and “slightly disagree” for the “powerful others” subscale means that they generally did not believe in the role of others in the maintenance of their own health.

**Table 1:** Descriptive statistics (listwise: valid n=718).

	Values, mean (SD; range)
Age (years)	46.41 (10.143; 21-68)
Gender	1 (0; 1-1)
Education	2.89 (1.398; 1-6)
COHL^a^1^b^	1.84 (0.763; 1-3)
COHL2^c^	1.91 (0.751; 1-3)
COHL3^d^	1.95 (0.744; 1-3)
eHEALS^e^1^f^	2.73 (1.241; 1-5)
eHEALS2^g^	2.69 (1.201; 1-5)
eHEALS3^h^	2.68 (1.158; 1-5)
eHEALS4^i^	2.86 (1.239; 1-5)
eHEALS5^j^	2.68 (1.2; 1-5)
eHEALS6^k^	2.78 (1.228; 1-5)
eHEALS7^l^	2.75 (1.203; 1-5)
eHEALS8^m^	2.84 (1.231; 1-5)
Internal scale^n^	18.05 (4.39; 6-31)
Chance scale^o^	16.8 (4.435; 6-30)
Doctor scale^p^	9.75 (3.333; 3-18)
Other people scale^q^	8.7 (2.757; 3-16)

^a^COHL: Communicative Health Literacy.

^b^“When you talk to a physician or nurse, do you give them all the information they need to help you?”

^c^“When you talk to a physician or nurse, do you ask the questions you need to ask?”

^d^“When you talk to a physician or nurse, do you ensure they explain anything that you do not understand?”

^e^eHEALS: eHealth Literacy Scale.

^f^“I know what health resources are available on the internet.”

^g^“I know where to find helpful health resources on the internet.”

^h^“I know how to find helpful health resources on the internet.”

^i^“I know how to use the internet to answer my health questions.”

^j^“I know how to use the health information I find on the internet to help me.”

^k^“I have the skills I need to evaluate the health resources I find on the internet.”

^l^“I can tell of high quality from low-quality health resources on the internet.”

^m^“I feel confident using information from the internet to make health decisions.”

^n^The internal locus of control: beliefs that one’s health is up to their own actions and behaviors.

^o^The chance locus of control: beliefs that one’s health is up to fate, chance, or luck.

^p^The doctor locus of control: beliefs that one’s health is up to doctors.

^q^The powerful others locus of control: beliefs that one’s health is up to others’ actions and behaviors.

### Model Fit Statistics

**Figure 1:**
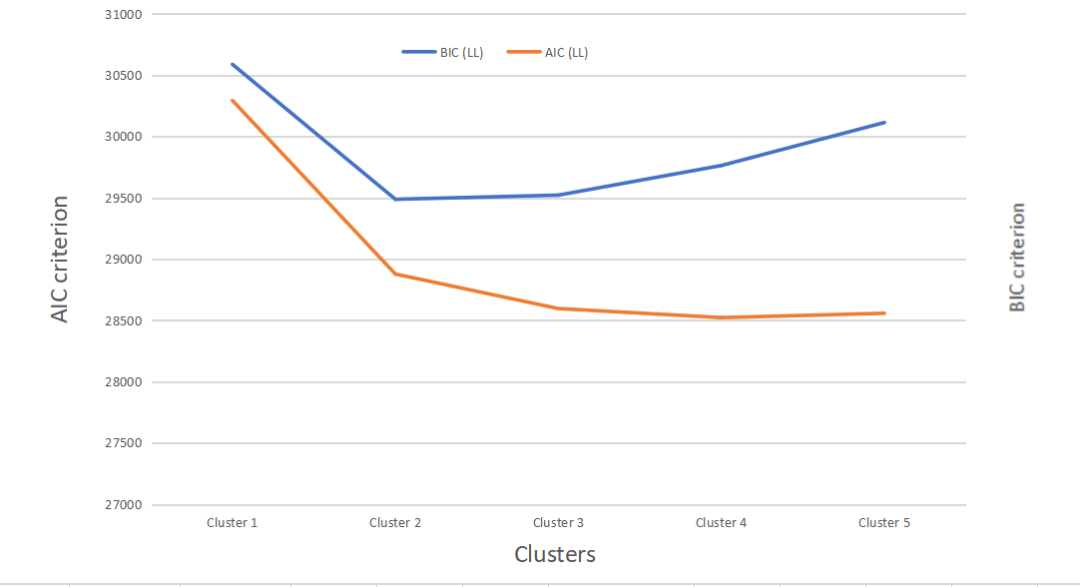
Model fit statistics changes (male participants’ data). AIC: Akaike information criterion; BIC: Bayesian information criterion; LL: log-likelihood.

Table 2 and Figure 1 show the model fit statistics of the LCA. The Akaike information criterion and Bayesian information criterion provide the measures of model performance. Smaller Akaike information criterion and Bayesian information criterion values indicate better model performance. Indexes such as the Lo-Mendell-Rubin likelihood ratio test and the bootstrap likelihood ratio test examined whether adding clusters would significantly improve model performance. We considered all the factors and decided to opt for a 2-cluster solution for better model performance and simplicity to guide subsequent qualitative analyses, as shown in Multimedia Appendix 3.

**Table 2:** Model fit statistics for male data.

	Number of latent classes	BIC^a^ (LL^b^)	AIC^c^ (LL)	AIC3 (LL)	Npar^d^	Maximum BVR^e^	Classification errors	Entropy *R*^2f^
Model1	Cluster 1	30588.8978	30293.9984	30365.9984	72	202.584	0	1
Model2	Cluster 2	29487.7888	28877.5109	29026.5109	149	20.9173	0.0023	0.9871
Model3	Cluster 3	29521.672	28596.0156	28822.0156	226	24.6777	0.0021	0.9901
Model4	Cluster 4	29763.3869	28522.3521	28825.3521	303	22.1771	0.006	0.9778
Model5	Cluster 5	30114.1405	28557.7272	28937.7272	380	20.8918	0.0241	0.9365

^a^BIC: Bayesian information criterion.

^b^LL: log-likelihood (the smaller the absolute value of LL, the better the model fit).

^c^AIC: Akaike information criterion.

^d^Npar: number of estimated parameters.

^e^BVR: bivariate residual.

^f^Entropy *R*^2^: values >0.8 indicate high degree of separation between classes.

### Profiling of 2 Latent Clusters

#### Cluster 1—Low Self-Efficacy Group

Male patients in cluster 1 had low scores on the “internal” scale (with conditional probabilities higher than 0.5 until an internal sum score of 22), suggesting limited self-efficacy and less inclination to believe in their own capability to manage self-health. For example, they were more likely to “strongly disagree” (coding 1), “moderately disagree” (coding 2), or “slightly disagree” (coding 3) with statements such as “If my condition worsens, it is my own behavior which determines how soon I will feel better again” (MHLC_C1), “I am directly responsible for my condition getting better or worse” (MHLC_C6), and “Whatever goes wrong with my condition is my own fault” (MHLC_C8).

Male patients in cluster 1 also ranked low on the “doctor” subscale (with conditional probabilities higher than 0.5 until the internal sum score of 11), suggesting that they had limited trust in medical and health professionals and the health benefits of adhering to their recommendations and advice. For example, they were more likely to “strongly disagree” (coding 1), “moderately disagree” (coding 2), or “slightly disagree” (coding 3) with statements such as “If I see my doctor regularly, I am less likely to have problems with my condition” (MHLC_C3).

Male patients in cluster 1 had low (score=4-9) to medium (score=10-14) scores on the “other people” subscale, suggesting that most people in cluster 1 were not likely to see the influence of others’ behaviors on their own health as significant. For example, they were more likely to “strongly disagree” (coding 1), “moderately disagree” (coding 2), or “slightly disagree” (coding 3) with statements such as “Other people play a big role in whether my condition improves, stays the same, or gets worse” (MHLC_C7), “In order for my condition to improve, it is up to other people to see that the right things happen” (MHLC_C10), and “The type of help I receive from other people determines how soon my condition improves” (MHLC_C18).

Male patients in cluster 1 had more spread scores across the “chance” subscale ranging from 12 to 28, suggesting that although some people in this cluster did not believe in the role of luck in one’s health management, others did agree with statements such as “As to my condition, what will be will be” (MHLC_C2), “Most things that affect my condition happen to me by chance” (MHLC_C4), “Luck plays a big part in determining how my condition improves” (MHLC_C9), “Whatever improvement occurs with my condition is largely a matter of good fortune” (MHLC_C11), “If my condition worsens, it’s a matter of fate” (MHLC_C15), and “If I am lucky, my condition will get better” (MHLC_C16).

#### Cluster 2—Moderate Self-Efficacy Group

Male patients in cluster 2 had high scores on the “internal” scale ranging from 23 to 31, suggesting that they had stronger beliefs in their own capability to manage their health. Their responses to the questions of the “internal” scale were more likely to “slightly agree,” “moderately agree,” or “strongly agree” with statements of the MHLC Scale, stressing the importance of self-discipline and self-management to achieve optimal health outcomes, especially regarding the prevention and treatment of bladder cancer. For example, people in cluster 2 agreed with statements such as “the main thing which affects my condition is what I myself do” (MHLC_C12), “I deserve the credit when my condition improves and the blame when it gets worse” (MHLC_C13), and “If my condition takes a turn for the worse, it is because I have not been taking proper care of myself” (MHLC_C17).

Male patients in cluster 2 had higher scores on the “doctor” subscale, ranging from 12 to 18, suggesting that they had moderate to high levels of trust in health and medical professionals and the importance of adherence to their advice to achieve better health outcomes. Similar to the “internal” subscale, their responses to the questions of the “doctor” subscale were more likely to “moderately agree” or “strongly agree” with statements of the MHLC Scale, highlighting the importance of seeking medical support to prevent, diagnose, and treat diseases. For example, “Whenever my condition worsens, I should consult a medically trained professional.” (MHLC_C5) and “Following doctor’s orders to the letter is the best way to keep my condition from getting any worse” (MHLC_C14).

Male patients in cluster 2 had very high (score=15-16) scores on the “other people” subscale, suggesting that some of them were more likely to associate their own health outcomes with other people in their lives.

Chinese male participants in cluster 2 were divided on the “chance” subscale, with some people having very low (score=6-11) scores and others having very high (score=28-30) scores. This indicated that a polarized view regarding the role of chance in their health and well-being existed among this group of male patients, despite them being internally driven and having stronger beliefs in medical professionals.

Table 3 shows descriptive statistics of the 2 latent clusters representing the 3 levels of self-efficacy among the study participants. The low self-efficacy group (cluster 1) represented 75.8% (544/718) of the total sample. They had an average of 17.52 (SD 3.66) on the internal scale, an average of 17.84 (SD 3.83) on the “chance” scale, an average of 8.29 (SD 2.36) on the “doctor” scale, and an average of 8.73 (SD 2.65) on the “other people” scale. The moderate self-efficacy group (cluster 2) represented 24.2% (174/718) of the total sample. They had an average of 19.36 (SD 5.39) on the “internal” scale, an average of 15.22 (SD 5.06) on the “chance” scale, an average of 13.01 (SD 2.56) on the “doctor” scale, and an average of 9.13 (SD 3.21) on the “other people” scale.

Next, we compared the differences between the 2 clusters across the 4 subscales. The result of the Welch test in Table 4 shows that there were statistically significant differences among the 2 clusters representing 2 distinct levels of self-efficacy among the study participants in their scores on the internal, “chance,” and “doctor” subscales but not on the “other people” subscale.

**Table 3:** Descriptive statistics of the latent clusters (N=718).

Subscale and cluster			Values, n (%)		Values, mean (SD)		Values, SE
**Internal scale**							
	1^a^	544 (75.8)		17.52 (3.66)		0.16	
	2^b^	174 (24.2)		19.36 (5.39)		0.41	
	Total	718 (100)		17.96 (4.21)		0.16	
**Chance scale**							
	1^a^	544 (75.8)		17.84 (3.83)		0.16	
	2^b^	174 (24.2)		15.22 (5.06)		0.38	
	Total	718 (100)		17.2 (4.31)		0.16	
**Doctor scale**							
	1^a^	544 (75.8)		8.29 (2.36)		0.10	
	2^b^	174 (24.2)		13.01 (2.56)		0.19	
	Total	718 (100)		9.43 (3.14)		0.12	
**Other people scale**							
	1^a^	544 (75.8)		8.73 (2.65)		0.11	
	2^b^	174 (24.2)		9.13 (3.21)		0.24	
	Total	718 (100)		8.83 (2.8)		0.10	

^a^Low self-efficacy.

^b^Moderate self-efficacy.

**Table 4:** Robust tests of equality of means (Welch Test).

	Statistic	*df*1	*df*2	*P* value
Internal scale	17.67	1.00	226.10	<.001
Chance scale	39.46	1.00	239.76	<.001
Doctor scale	464.03	1.00	273.01	<.001
Other people scale	2.18	1.00	252.34	.14

### Factors Associated With Low Self-Efficacy

#### Limited Educational Attainment

Table 5 presents the conditional probabilities of educational attainment within the 2 identified clusters of self-efficacy. It can be seen that male patients with low self-efficacy were more likely to have lower levels of educational attainment (Year 6 to Year 12). In contrast, male patients with moderate self-efficacy were more likely to have adequate education (diploma and university).

**Table 5:** Conditional probabilities of educational levels within each cluster.

Education	Cluster 1^a^	Cluster 2^a^
Year 6	0.24	0.05
Year 9	0.28	0.15
Year 12	0.20	0.21
Diploma	0.20	0.23
University	0.07	0.27
Postgraduate	0.00	0.10

^a^Total probability: 1.00.

#### Aged ≥44 Years

Table 6 shows the conditional probabilities of age groups within the 2 identified clusters of self-efficacy. This table shows that male patients with low self-efficacy were more likely to be middle-aged and older (≥44 years). In contrast, male patients with moderate self-efficacy were more likely to be aged ≤43 years.

**Table 6:** Conditional probabilities of age groups within each cluster.

Age group (years)	Cluster 1^a^	Cluster 2^a^
21-36	0.15	0.41
37-43	0.17	0.20
44-49	0.20	0.15
50-56	0.25	0.14
57-68	0.23	0.10

^a^Total probability: 1.00.

#### Limited Communicative Health Literacy

Table 7 shows that male patients with low self-efficacy were less likely to share with medical professionals all the information doctors need during medical encounters. In contrast, male patients of adequate self-efficacy were more likely to “often” share with medical professionals all the information doctors need during medical encounters.

**Table 7:** Conditional probabilities of responses to the first question of Communicative Health Literacy (COHL) within each cluster.

COHL1^a^	Cluster 1^b^	Cluster 2^b^
Often	0.31	0.59
Sometimes	0.40	0.38
Rarely	0.29	0.03

^a^“When you talk to a doctor or nurse, do you give them all the information they need to help you?”

^b^Total probability: 1.00.

#### Limited Digital Health Literacy

Table 8 shows that male patients with low self-efficacy were less likely to know where to find useful information on the internet. Table 9 shows that male patients with low self-efficacy were less likely to know the means and methods to identify useful health information on the internet. Male patients with low self-efficacy were more likely to disagree or feel unsure that they had the skills and knowledge that enabled them to navigate eHealth platforms and find helpful health-related information. In contrast, male patients with adequate self-efficacy were more likely to feel unsure or agree that they were equipped with such essential skills and knowledge.

**Table 8:** Conditional probabilities of responses to the second question of eHealth Literacy Scale (eHEALS) within each cluster.

eHEALS2^a^	Cluster 1^b^	Cluster 2^b^
Strongly disagree	0.24	0.05
Disagree	0.30	0.13
Unsure	0.28	0.46
Agree	0.10	0.25
Strongly agree	0.09	0.12

^a^“I know where to find useful health information on the internet.”

^b^Total probability: 1.00.

**Table 9:** Conditional probabilities of responses to the third question of eHealth Literacy Scale (eHEALS) within each cluster.

eHEALS3^a^	Cluster 1^b^	Cluster 2^b^
Strongly disagree	0.22	0.05
Disagree	0.33	0.12
Unsure	0.25	0.45
Agree	0.12	0.31
Strongly agree	0.07	0.07

^a^“I know how to find useful information on the internet.”

^b^Total probability: 1.00.

## Discussion

### Principal Findings in Relation to Previous Studies

We identified 2 subgroups—low and moderate self-efficacy groups—which represented 75.8% (544/718) and 24.2% (174/718) of the total sample, respectively. People in the low self-efficacy cluster (cluster 1: 544/718, 75.8%) had the following characteristics: they had low posterior probabilities on the “internal” and “doctor” subscales, suggesting that they were less likely to believe in their own capability or doctors’ advice to achieve optimal outcomes in bladder cancer prevention and treatment; they had higher posterior probabilities on the low (4-9) to medium (10-14) sections of the “other people” subscale, suggesting that most people in cluster 1 were not likely to see the influence of others’ behaviors on their health as significant; and finally, male patients in the low self-efficacy cluster had mixed beliefs about the role of chance and good luck on their health outcomes, as indicated by the wide-ranging sum scores on the “chance” subscale. Male patients in the moderate self-efficacy cluster (cluster 2: 174/718, 24.2%) had distinct psychological traits. They had high scores on the “internal” subscale, suggesting that they had stronger beliefs in their own capability to manage their health. They also had higher scores on the “doctor” subscale, suggesting that they had moderate to high levels of trust in health and medical professionals and the importance of adherence to their advice to achieve better health outcomes. Surprisingly, they had very high (score=15-16) scores on the “other people” subscale, suggesting that some of them were more likely to associate their own health outcomes with other people in their lives. Finally, we found that a polarized view regarding the role of chance in health and well-being existed among this group of male patients, despite being internally driven and having stronger beliefs in medical professionals.

The characteristics of the 2 clusters mentioned above could not be compared with relevant findings reported in previous studies because of the lack of relevant literature. However, we could relate the factors associated with the low-efficacy cluster identified in our study to relevant factors correlated with cancer risk, view of cancer, and cancer prevention, ascertained in a limited number of studies. As revealed in our study, limited communicative and digital health literacy contributed to low self-efficacy, that is, being less likely to believe in one’s own capability or doctors’ advice to achieve optimal outcomes in bladder cancer prevention and treatment. This finding aligns well with that reported by Morris et al [40] that those with low health literacy tended to feel less control over risks to their health and take more fatalistic attitudes toward cancer and cancer prevention, thus avoiding visiting doctors and being less likely to be up-to-date on cancer screening. The high-efficacy cluster in our study, in contrast, preferred to seek cancer prevention information from others and web-based resources, suggesting that they were more likely to believe in their own abilities, doctors’ recommendations, or others’ behaviors to achieve optimal outcomes in bladder cancer prevention and treatment. This finding somewhat confirms that of Morris et al [40].

In our study, we found that being aged ≥44 years was one indicator of participants’ low self-efficacy in bladder cancer prevention and treatment, which supports the findings by Taber et al [41] and Katapodi et al [42] that older age was correlated with lower perceived cancer risk, inducing older adults to take relatively negative attitudes toward cancer prevention and treatment. Similarly, Schroyen et al [43] reported that self-perception of aging and views of cancer could be seen as markers of vulnerability among older people with cancer. Similarly, the report by Schroyen et al [43] implied that older age and attitude toward cancer were correlated with low self-efficacy. Deeks et al [44] found that older study participants regarded a stable home life and relationships as important factors influencing health, which is contrary to our finding that older age was closely associated with low self-efficacy with regard to bladder cancer prevention and treatment, as shown by their perception of others’ behaviors as insignificant in influencing their health. Deeks et al [44] also discovered that having a disease prevention strategy was perceived as one of the most influential factors impacting health compared with our finding that patients in the low self-efficacy cluster displayed negative beliefs in their internal motivations to engage in health improvement.

Leung et al [45] found that fatalistic beliefs were negatively associated with cancer-related information-seeking behaviors, that is, patients who viewed the role of chance and good luck in their health outcomes as significant were less willing to seek information from health professionals and media. This finding supports our finding that patients in the low self-efficacy cluster were unlikely to regard the impact of others on their health as significant.

We identified low educational attainment as a significant contributor to low self-efficacy, specifically to negative attitudes toward the “internal,” “doctor,” or “others” LOC. However, we did not find such an association in the literature. This warrants further studies to ascertain the role of education, as “there exists a strong educational gradient in cancer risk, which has been documented in a wide range of populations” [46].

### Implications

This study has some implications for clinical practice, health education, medical research, and public health policy-making. The 2 self-efficacy classes and 4 factors contributing to low self-efficacy can serve as important indicators for screening male patients with low self-efficacy to deliver more targeted education and more effective interventions to enhance their self-efficacy. Knowledge, skills, beliefs, and practices associated with the low self-efficacy class and the contributing factors could be integrated into public health education about and interventions in health beliefs about bladder cancer prevention and treatment among male patients to enhance their self-efficacy. Medical researchers can gain some insights into the topic of low self-efficacy and the contributing factors. Informed by this study, they could identify patients with low self-efficacy among their ethnic and socioeconomic groups, verify the contributing factors ascertained in this study, and identify more contributors in future studies. Finally, our research results and findings can provide some implications for public health policy-making in the future.

### Limitations

This study has some limitations. First, the generalizability of our results and findings may be limited. The recruitment of patients from only 1 hospital was most likely to make the results and findings less generalizable to populations in other provinces in China and to patients in different linguistic and cultural communities worldwide. Further research is warranted to validate the results and findings among populations of diverse ethnic and sociocultural backgrounds. Second, the self-reported nature of the collected data may result in some bias. As claimed by van der Varrt et al [47], self-reported literacy skills are not necessarily consistent with the actual abilities to comprehend, use, and appraise web-based health information. This is true for the self-reported literacy skills on the functional subscale of the AAHLS and the self-reported health beliefs and self-confidence on the MHLC Form C used in this study. More objective measures need to be developed to increase the reliability and consistency of assessments of various health literacy and health beliefs and self-confidence among culturally and linguistically diverse people. Third, comparisons could not sufficiently be made with previous studies because of the scarcity of relevant literature. Hopefully, our study will attract close attention from researchers who can further examine this topic to add to the body of literature and expand knowledge, which could promote academic conversation around such a topic of social significance.

### Conclusions

This was the first study to investigate the attitudes and beliefs regarding bladder cancer treatment among male patients in China. Using latent class modeling, we identified 2 classes of LOC groups among male patients, the low self-efficacy group and the moderate self-efficacy group. Four factors contributing to low self-efficacy were identified, including (1) limited education (Year 6 to Year 12), (2) aged ≥44 years, (3) limited communicative health literacy, and (4) limited digital health literacy. These contributing factors can provide some implications for clinical practice, health education, medical research, and health policy-making.

## Appendix

Multimedia Appendix 1 Questionnaire (Chinese).

Multimedia Appendix 2 Data collected and coding method (Chinese).

Multimedia Appendix 3 Table S1: Posterior probabilities of response across latent clusters (male participants).

## Abbreviations

AAHLS: All Aspects of Health Literacy Scale
eHEALS: eHealth Literacy Scale
LCA: latent class analysis
LOC: locus of control
MHLC: Multidimensional Health Locus of Control
