# Supplementary material for: Using the Multidimensional Health Locus of Control Scale Form C to Investigate Health Beliefs About Bladder Cancer Prevention and Treatment Among Male Patients: Cross-Sectional Study
Source: JMIR Form Res. 2023 Aug 16;7:e43345. doi: 10.2196/43345 (PMC10468698; doi:10.2196/43345)
Supplement: Multimedia Appendix 3 [file formative_v7i1e43345_app3.docx]

**Table S1.** Posterior probabilities of response across latent clusters (male participants).

| Scales and overall values | | Cluster 1 | Cluster 2 |
| --- | --- | --- | --- |
| **Internal subscale^a^** | | | |
|  | 6 | 0 | 1 |
|  | 8 | 0 | 1 |
|  | 9 | 0 | 1 |
|  | 10 | 0.55 | 0.45 |
|  | 11 | 0.75 | 0.25 |
|  | 12 | 0.85 | 0.15 |
|  | 13 | 0.87 | 0.13 |
|  | 14 | 0.81 | 0.19 |
|  | 15 | 0.92 | 0.08 |
|  | 16 | 0.83 | 0.17 |
|  | 17 | 0.84 | 0.16 |
|  | 18 | 0.88 | 0.12 |
|  | 19 | 0.89 | 0.11 |
|  | 20 | 0.82 | 0.18 |
|  | 21 | 0.87 | 0.13 |
|  | 22 | 0.7 | 0.3 |
|  | 23 | 0.38 | 0.62 |
|  | 24 | 0.37 | 0.63 |
|  | 25 | 0.46 | 0.54 |
|  | 26 | 0 | 1 |
|  | 27 | 0.49 | 0.51 |
|  | 28 | 0.5 | 0.5 |
|  | 29 | 0 | 1 |
|  | 30 | 0 | 1 |
|  | 31 | 0 | 1 |
| **Chance subscale^b^** | | | |
|  | 6 | 0 | 1 |
|  | 7 | 0 | 1 |
|  | 8 | 0.17 | 0.83 |
|  | 9 | 0.25 | 0.75 |
|  | 10 | 0.5 | 0.5 |
|  | 11 | 0.41 | 0.59 |
|  | 12 | 0.63 | 0.37 |
|  | 13 | 0.76 | 0.24 |
|  | 14 | 0.74 | 0.26 |
|  | 15 | 0.83 | 0.17 |
|  | 16 | 0.81 | 0.19 |
|  | 17 | 0.9 | 0.1 |
|  | 18 | 0.82 | 0.18 |
|  | 19 | 0.85 | 0.15 |
|  | 20 | 0.94 | 0.06 |
|  | 21 | 0.86 | 0.14 |
|  | 22 | 0.93 | 0.07 |
|  | 23 | 0.79 | 0.21 |
|  | 24 | 0.73 | 0.27 |
|  | 25 | 0.8 | 0.2 |
|  | 26 | 1 | 0 |
|  | 27 | 1 | 0 |
|  | 28 | 0.33 | 0.67 |
|  | 29 | 0 | 1 |
|  | 30 | 0 | 1 |
| **Doctor subscale^b^** | | | |
|  | 3 | 0.50 | 0.50 |
|  | 4 | 1.00 | 0.00 |
|  | 5 | 1.00 | 0.00 |
|  | 6 | 1.00 | 0.00 |
|  | 7 | 0.96 | 0.04 |
|  | 8 | 1.00 | 0.00 |
|  | 9 | 0.90 | 0.10 |
|  | 10 | 0.92 | 0.08 |
|  | 11 | 0.92 | 0.08 |
|  | 12 | 0.48 | 0.52 |
|  | 13 | 0.41 | 0.59 |
|  | 14 | 0.29 | 0.71 |
|  | 15 | 0.00 | 1.00 |
|  | 16 | 0.00 | 1.00 |
|  | 17 | 0.00 | 1.00 |
|  | 18 | 0.00 | 1.00 |
| **Other people subscale^b^** | | | |
|  | 4 | 0.60 | 0.40 |
|  | 5 | 0.79 | 0.21 |
|  | 6 | 0.68 | 0.32 |
|  | 7 | 0.78 | 0.22 |
|  | 8 | 0.85 | 0.15 |
|  | 9 | 0.77 | 0.23 |
|  | 10 | 0.91 | 0.09 |
|  | 11 | 0.83 | 0.17 |
|  | 12 | 0.59 | 0.41 |
|  | 13 | 0.73 | 0.27 |
|  | 14 | 0.62 | 0.38 |
|  | 15 | 0.33 | 0.67 |
|  | 16 | 0.50 | 0.50 |

^a^Overall: cluster 1—0.75; cluster 2—0.25.

^b^Overall: cluster 1—0.7514; cluster 2—0.2486.
